# Activation of PD-1/PD-L1 immune checkpoint by Zika virus

**DOI:** 10.1371/journal.ppat.1013457

**Published:** 2025-09-08

**Authors:** Chenxi Wang, Yubin Xie, Weixin Li, Chon Phin Ong, Hao Ding, Shuofeng Yuan, Gong Cheng, Dong-Yan Jin, Zi-Wei Ye

**Affiliations:** 1 School of Biomedical Sciences, LKS Faculty of Medicine, The University of Hong Kong, Pokfulam, Hong Kong, China; 2 Department of Microbiology and State Key Laboratory of Emerging Infectious Diseases, LKS Faculty of Medicine, Pokfulam, Hong KongChina; 3 Tsinghua-Peking Center for Life Sciences, School of Medicine, Tsinghua University, Beijing, China; University of Minnesota Twin Cities, UNITED STATES OF AMERICA

## Abstract

Zika virus (ZIKV) has emerged as a rising concern in global health in recent years. The role of PD-1/PD-L1 immune checkpoint in acute ZIKV infection remains to be understood. In this study we demonstrated the activation of PD-1/PD-L1 immune checkpoint by ZIKV. mRNA and protein expression of PD-L1 was boosted by ZIKV not only in SF268 and JEG3 cell lines but also in human dendritic cells. PD-1 expression was more abundant on CD8^+^ T cells in ZIKV-infected mice. Elevated PD-L1 expression was also observed in the brain, testis and spleen of ZIKV-infected A129 mice. Blocking PD-L1 effectively inhibited ZIKV infection, reducing viral loads in all tissues. In addition, anti-PD-L1 antibody treatment further increased virus-specific CD8^+^ T cells, KLRG^+^ CD8^+^ T cells, and effector memory CD8^+^ T cells. PD-L1 blockade also induced interferon γ, granzyme B, and interleukin 2 expression in antigen-specific CD8^+^ T cells, consistent with activation of these cells. Mechanistically, the induction of PD-L1 expression might be ascribed to viral NS4B protein and its interaction with GRP78. Our findings suggest that targeting the PD-1/PD-L1 pathway could have antiviral effect against ZIKV.

## Author summary

Immune checkpoint blockade has become another pillar in cancer treatment, but whether the same strategy might be used in antiviral therapy remains to be clarified. In this study we used ZIKV infection as an example to understand immune checkpoint activation during acute infection. The activation of the PD-1/PD-L1 immune checkpoint by ZIKV was demonstrated by several lines of evidence. Blocking this checkpoint using anti-PD-L1 antibodies effectively inhibited ZIKV infection in mice, reducing viral burdens in all tissues, consistent with enhanced virus-specific T cell response. It was further found that the ability to induce PD-L1 expression would be ascribed to one viral protein named NS4B and its interaction with another cellular protein GRP78. Our work revealed a new antiviral strategy against ZIKV by blocking the PD-1/PD-L1 immune checkpoint.

## Introduction

Zika virus (ZIKV) has raised fresh concerns in global health in recent years. Since its discovery in Uganda in 1947, this mosquito-borne virus has spread to 89 countries worldwide. ZIKV is primarily transmitted to humans through the bite of an infected *Aedes* mosquito. It can also be sexually transmitted, leading to testicular damage and prolonged presence in semen and vaginal secretions [1]. While most individuals infected with ZIKV experience mild symptoms such as fever, rash, conjunctivitis, and muscle pain, the virus poses a severe risk during pregnancy by crossing the placenta to cause birth defects like microcephaly and congenital malformations in fetuses and newborn infants [2]. Additionally, ZIKV could also cause Guillain-Barré syndrome (GBS) and meningoencephalitis in infected adults [3]. The lack of vaccines or antivirals poses an urgent need to advance our understanding of the biology and immunology of ZIKV infection.

Both innate and adaptive immune systems play pivotal roles in the host response against ZIKV infection [4,5]. The interferon (IFN) system serves as a critical host innate antiviral defense. Elevated levels of IFN-α, IFN-β, TLR3, RIG-I and other innate immune mediators have been reported in patients infected with ZIKV [6]. The expression of type I IFN-stimulated genes such as IFITM1 and IFITM3 also inhibits ZIKV infection [7]. To evade the host’s antiviral response and facilitate replication, ZIKV has developed various evasion strategies [8]. Like other viruses in the *Flaviviridae* family, the ZIKV genome is translated into a single polyprotein, which is subsequently cleaved into three structural proteins — capsid, pre-membrane (prM) and envelope (E), as well as seven non-structural (NS) proteins: NS1, NS2A, NS2B, NS3, NS4A, NS4B and NS5. ZIKV employs its multiple NS proteins to antagonize IFN response [9]. Particularly, the NS1 protein stabilizes caspase 1 to cleave cGAS, which is activated by mitochondrial DNA during ZIKV infection [10]. The NS2A protein facilitates degradation of STAT1 and STAT2 [11]. The NS2B-NS3 protease of ZIKV is known to cleave STING [12,13], while the NS3 helicase targets RIG-I, MDA5 and MAVS [13,14]. The NS4A protein disrupts the interaction between RIG-I and MAVS [15], whereas NS4B inhibits STAT1 phosphorylation [16] and TBK1 activation [17]. Notably, STAT2 is more specifically targeted by the NS5 protein [18,19]. Unlike dengue virus (DENV), the NS5 protein of ZIKV utilizes the ZSWIM8–CUL3 E3 ligase complex instead of UBR4 to degrade STAT2 [20]. As such, ZIKV inhibits both STAT1 and STAT2 phosphorylation in primary human dendritic cells to antagonize type I IFN signaling [21].

In addition to innate immune response, the importance of adaptive immunity in host defense against ZIKV has also been highlighted [5,22]. T cell activation in response to ZIKV infection is rather robust in both human and mouse models [8]. Immunocompetent mice exposed to ZIKV exhibit a strong CD8^+^ T cell response [23,24], which have both protective and cytotoxic effects [25,26]. Whereas the viral loads are reduced upon adoptive transfer of ZIKV-immune CD8^+^ T cells, depletion of these cells results in higher viral burdens in tissues. In addition, ZIKV infection was more lethal in CD8^-/-^ mice [8]. In mice treated with anti-IFNAR antibodies without T cell depletion, body weight loss was not observed despite increased viral replication, suggesting that T cells play a crucial role in limiting ZIKV infection specifically when type I IFN response is compromised [27]. In another model using IFNAR KO mice, depletion of CD8^+^ T cells led to higher viral loads in the brain but resulted in improved survival rate and reduced paralysis [28]. Interestingly, an epidemic Brazilian isolate of ZIKV from the outbreak potently suppressed CD8^+^ T cell immunity in mice by perturbing antigen presentation [29]. However, additional mechanisms by which ZIKV counteracts adaptive immunity remains to be further elucidated.

Programmed cell death receptor 1 (PD-1), a crucial inhibitory receptor in the CD28 superfamily, is specifically expressed in activated T cells. Its ligand PD-L1 is broadly expressed in various cell types, including antigen-presenting cells, epithelial cells and endothelial cells. Interaction between PD-1 and PD-L1/2 impedes T cell receptor signaling, leading to the suppression of T cell expansion and function [30]. As such, immune checkpoint therapy based on PD-1/PD-L1-blocking antibodies has emerged as a new pillar of cancer therapy [30,31]. Notably, the upregulation of PD-1/PD-L1 during acute virus infection also results in the suppression of T cell response [32]. For example, in mice infected with acute lymphocytic choriomeningitis virus (LCMV), PD-1 expression rapidly increases upon activation of naive virus-specific CD8^+^ T cells within 24 hours post-infection. Blocking the PD-1 pathway using anti-PD-L1 or anti-PD-1 antibodies during the early phase of acute LCMV infection results in accelerated clearance of the virus [33]. Likewise, metapneumovirus and influenza A also upregulate PD-1 and PD-L1 to impair CD8^+^ T cells response [34]. In addition, in infection with high-pathogenicity influenza virus, PD-1 expression on virus-specific CD8^+^ T cells elevates, and *in vivo* blockade of PD-L1 leads to decreased viral replication and increased CD8^+^ T cell function [35]. Whereas the lack of PD-1 restores CD8^+^ T cell function in mice [34], genetic knockout of PD-L1 in mice does not restore CD8^+^ T cell function and delays viral clearance [36]. Although ZIKV has been shown to sensitize glioblastoma cells to anti-PD-L1 immunotherapy [37], further investigations are required to clarify exactly how ZIKV affects the PD-1/PD-L1 immune checkpoint.

We report here that ZIKV infection leads to the upregulation of PD-L1 both in cultured cells and *in vivo*. Remarkably, blocking PD-L1 effectively suppressed ZIKV infection in A129 mice that are deficient of type I IFN signaling. Treatment with anti-PD-L1 antibodies enhanced the response of virus-specific CD8^+^ T cells. The NS4B protein and its interaction partner GRP78 were identified in the functional screens for proteins that induce PD-L1 expression. Targeting the PD-1/PD-L1 pathway holds the promise to be another strategy in antiviral development against ZIKV.

## Results

### ZIKV infection upregulates PD-L1 expression in cultured human cells

To shed light on the impact of ZIKV on host cells, we performed a comparative analysis of differentially expressed genes (DEGs) in ZIKV-infected JEG3 human choriocarcinoma cells (JEG-3), astrocytes (U-251 MG), and human renal proximal tubular epithelial cells (HK-2) using publicly available RNA-seq data [38]. A total of 92 DEGs were overlapped in all three cell lines (S1A Fig). Gene Ontology Biological Processes (GOBP) analysis revealed that most of these genes are associated with innate and adaptive immune responses as well as metabolic processes (S1B Fig). Among the genes related to adaptive immune response, PD-L1 was consistently upregulated in all three cell lines (S1C Fig). However, the functional relevance of PD-L1 upregulation in ZIKV infection has not been determined.

**Fig 1:**
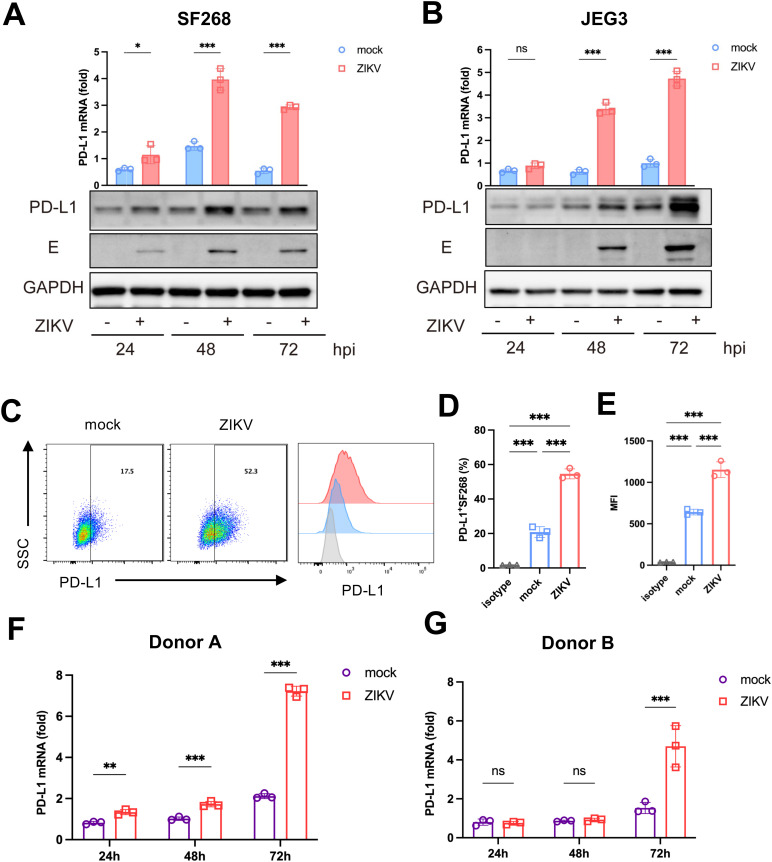
ZIKV infection upregulates PD-L1 expression in cultured cells. SF268 cells (**A**) and JEG3 cells (**B**) were infected with ZIKV strain PRVABC59 at a MOI of 1. Cell lysates were collected at 24, 48 and 72 hpi. The mRNA levels of PD-L1 were assessed using RT-qPCR. GAPDH was used as a housekeeping control. Data are presented as the mean ± SD of three independent experiments. Statistical analyses were performed by two-way ANOVA (*P < 0.05; ***P < 0.001). Protein levels of PD-L1 and envelope (E) protein were assessed by Western blotting with GAPDH as a loading control. Flow cytometric analysis of PD‐L1 in SF268 cells upon ZIKV infection at 48 hpi. SF268 cells were analyzed for the expression of PD-L1 (gated on live cells) **(C)**, quantification of the frequency of PD-L1 positive cells **(D)**, quantification of the mean fluorescence intensity (MFI) of PD-L1 **(E)**. The PD-L1 expression in ZIKV-infected human dendritic cells (DCs) from donor A (**F**) and donor B **(G)**. DCs were infected with ZIKV at an MOI of 1. The mRNA levels of PD-L1 were assessed using RT-qPCR. Data are presented as the mean ± SD of three independent experiments. Statistical analyses were carried out by two-way ANOVA (**P < 0.01; ***P < 0.001).

We verified the upregulation of PD-L1 during ZIKV infection. Human glioblastoma cell line SF268 was infected with ZIKV strain PRVABC59 at a multiplicity of infection (MOI) of 1 for 72 hours. The mRNA levels of PD-L1 increased as the viral infection progressed, peaking at 48 hours post-infection (hpi) (Fig 1A). Consistent with this, Western blot analysis indicated elevated PD-L1 protein levels at 48 and 72 hpi (Fig 1A). The same trend of PD-L1 upregulation at both mRNA and protein levels was also observed in ZIKV-infected JEG3 cells at 48 and 72 hpi (Fig 1B). Flow cytometric analysis of infected SF268 and JEG3 cells showed a marked increase in PD-L1-positive cells and enhanced PD-L1 staining as indicated by the mean fluorescence intensity (MFI). (Figs 1C-1E and S2A-S2C). Notably, the same pattern of PD-L1 upregulation was also observed with ZIKV Uganda strain in both SF268 and JEG3 cells (S3 Fig).

Dendritic cells (DCs) are the major PD-L1-expressing cells [31]. They are susceptible to ZIKV infection [21]. As professional antigen presenting cells, they play a pivotal role in the activation of T cell response against ZIKV [39]. We found that ZIKV infection induced PD-L1 expression in human DCs from different donors (Fig 1F and 1G). These results indicate the induction of PD-L1 in a relevant and important type of human cells by ZIKV. Collectively, our findings demonstrate that ZIKV infection leads to the upregulation of PD-L1 expression.

### ZIKV induces PD-L1 expression and T cell response *in vivo*

**Fig 2:**
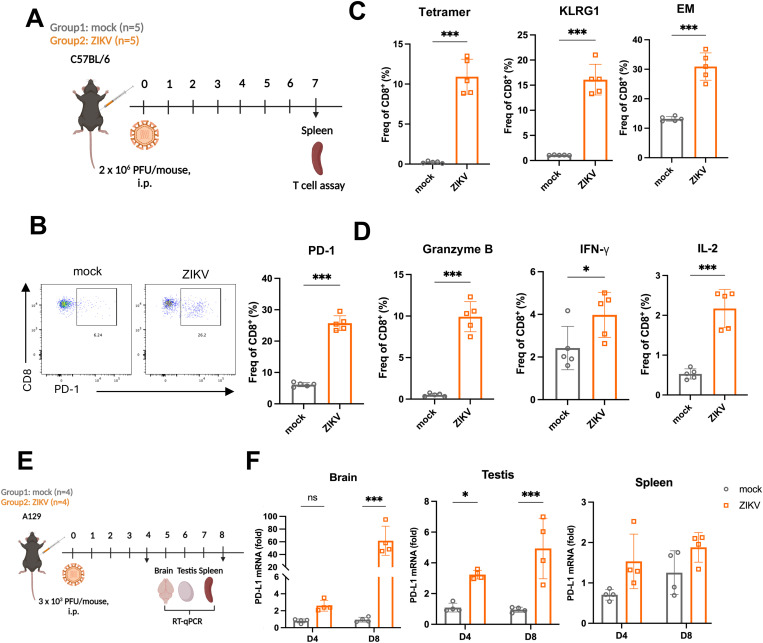
ZIKV infection induces T cell response and upregulates PD-L1 expression *in vivo.* **(A)** Viral challenge scheme for the C57BL/6 model. C57BL/B6 mice were intraperitoneally (i.p.) exposed to 2 × 10^6^ PFU of the virus or PBS (n = 5/group) at a final volume of 100 μL. Spleens collected on 7 days post-infection (dpi) were subjected to flow cytometric analysis. The figure was created using BioRender (https://BioRender.com). **(B)** Flow cytometric analysis of CD8^+^ activation upon ZIKV infection. Representative plots (left) and proportion of PD-1^+^ cells (right) are shown. **(C)** Quantification of the frequency (Freq) of tetramer^+^, KLRG1^+^, and effector memory (EM) CD8^+^ T cells. **(D)** Quantification of the frequency of granzyme B, IFN-γ and IL-2. Statistical analyses were performed by Student’s t-test (*P < 0.05; ***P < 0.001). **(E)** Viral challenge scheme for the A129 mice model. A129 mice were i.p. exposed to 3 × 10^3^ PFU of the virus or PBS (n = 4/group) at a final volume of 100 μL. The figure was created using Biorender (https://BioRender.com). **(F)** Brain, testis and spleen were collected on 8 dpi. Relative PD-L1 mRNA levels were detected using RT-qPCR analysis. Data are presented as the mean ± SD of three independent experiments. Statistical analyses were performed by two-way ANOVA (*P < 0.05; ***P < 0.001).

We went on to determine the functional outcome of PD-L1 upregulation in ZIKV-infected cells. ZIKV infection triggers robust CD8^+^ T cell response in C57BL/6 (B6) wild-type (WT) mice [23,24]. To confirm the activation of T cell response during ZIKV infection *in vivo*, B6 mice were intraperitoneally (i.p.) exposed to 2 × 10^6^ plaque-forming units (PFU) of the virus, and spleens were collected on 7 days post-infection (dpi) for analysis of T cell response (Fig 2A). An increase in PD-1 expression on CD8^+^ T cells was seen (Fig 2B). A ZIKV-specific tetramer was used to evaluate and quantify antigen-specific T cells. The number of ZIKV-specific CD8^+^ T cells in the spleens elevated (Fig 2C). In addition, KLRG1^+^ CD8^+^ effector T cells and effector memory T cells (Fig 2C) as well as CD8^+^ T cells expressing granzyme B, interleukin 2 (IL-2) and IFN-γ (Fig 2D) were more frequently detected in B6 mice following ZIKV infection, indicating the activation of CD8^+^ T cell response *in vivo*.

However, since B6 mice infected with ZIKV were asymptomatic except for a mild gain in body weight, further assessment of the pathogenicity of ZIKV infection in this model was not possible. The IFN-α/β receptor-deficient A129 mice have been utilized as a lethal model for ZIKV infection [40]. To confirm PD-L1 induction *in vivo*, A129 mice were i.p. infected with ZIKV, and the brain, testis and spleen were collected on 4 and 8 dpi (Fig 2E). An upregulation of PD-L1 mRNA expression was noted in these tissues, particularly in the brain on 8 dpi (Fig 2F), in support of the induction of PD-L1 expression by ZIKV *in vivo*.

### PD‐L1 blockade alleviates pathogenicity of ZIKV and elicits robust T cell response in mice

**Fig 3:**
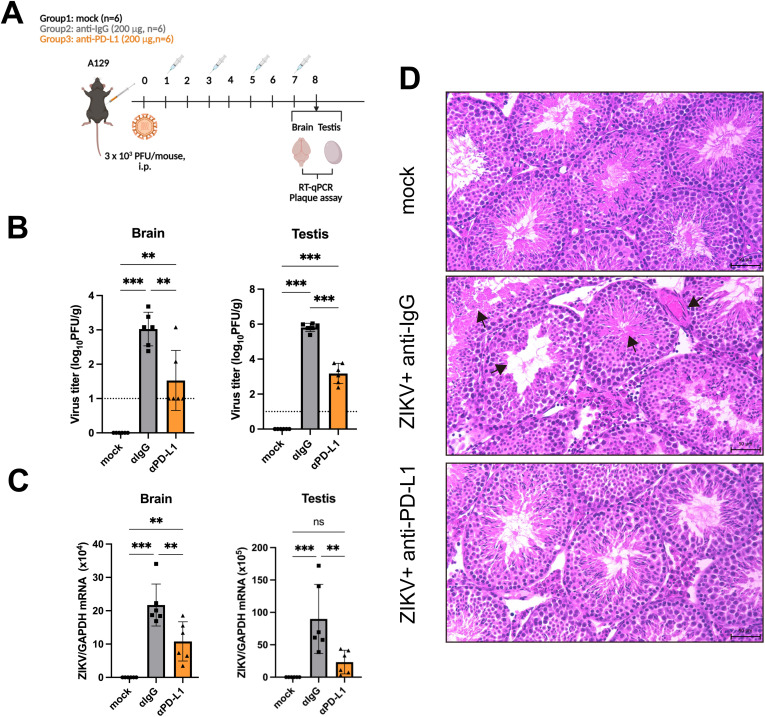
PD-L1 blockade alleviates pathogenicity of ZIKV in A129 mice. **(A)** Antibody treatment and viral challenge scheme for the A129 model. Mice were inoculated intraperitoneally (i.p.) with 3 × 10^3^ PFU of ZIKV at a final volume of 100 μL. Mice were i.p. treated with 200 μg isotype control (αIgG) or anti-PD-L1 antibody (αPD-L1) at a final volume of 100 μL on 1, 3, 5 and 7 dpi. Mock treatment was used as control. Mice were euthanized on day 8 post-virus challenge for sample harvest. The figure was created using BioRender (https://BioRender.com). **(B)** ZIKV viral titers in the brain and testis were determined using plaques assay (n = 6/group). The dashed line represents the limit of detection (LOD). **(C)** ZIKV mRNA levels in the brain and testis were determined using RT-qPCR by detecting the E gene (n = 6/group). Statistical analyses were performed by one-way ANOVA (*P < 0.05; **P < 0.01; ***P < 0.001). **(D)** Representative images of H&E staining of testis sections. Scale bars: 50 μm. Black arrows indicate tissue damage.

To interrogate the functional consequence of blocking PD-L1 in the context of ZIKV infection, male A129 mice were i.p. inoculated with ZIKV and subsequently treated with either an isotype control or a recombinant mouse monoclonal anti-PD-L1 antibody (Azeto) on 1, 3, 5 and 7 dpi (Fig 3A). Notably, treatment with the anti-PD-L1 antibody dampened the viral loads in the brain and testis (Fig 3B and 3C). Histopathological examination of the testis was performed using hematoxylin and eosin (H&E) staining to compare inflammation and tissue damage between the IgG and anti-PD-L1 groups. At 8 dpi, minimal damage was observed in the testis of the anti-PD-L1-treated group compared to the IgG-treated group (Fig 3D). To further evaluate the impact of PD-L1 blockade *in vivo*, male A129 mice were i. p. inoculated with 2 × 10³ PFU of ZIKV and subsequently treated with either 150 μg of either IgG control or anti-PD-L1 antibody on 2, 4, 6, and 8 dpi (S4A Fig). The anti-IgG-treated group showed significantly higher clinical scores than the anti-PD-L1 group at 4–6 dpi, including reduced activity, pronounced hunching, and hind limb paralysis (S4B Fig). Mice receiving the IgG control antibody showed a survival rate of 50%, whereas the anti-PD-L1-treated group had a higher survival rate of 62.5% (S4C Fig). However, this difference was not statistically significant.

To formally exclude the possibility that either the inhibition of PD-L1 or the anti-PD-L1 antibody used in the *in vivo* study has an unrecognized effect to result in the observed phenotypes, we asked whether PD-L1 blockade in cultured cells might affect ZIKV infection. Knockdown of PD-L1 expression in ZIKV-infected U251 cells with two independent siRNAs had no influence on ZIKV E protein expression (S5A Fig) or viral titer (S5B Fig). Consistent with this, E protein expression remained constant when PD-L1 expression was progressively increased (S5C Fig). In addition, ZIKV viral titers remained unchanged in cultured SF268 and U251 cells treated with the same anti-PD-L1 monoclonal antibody (Azeto) used in our *in vivo* studies (S5D Fig), suggesting that it did not exert any unexpected effect on ZIKV replication. Since this antibody directed against mouse PD-L1 might not recognize human PD-L1 optimally or cross the plasma membrane, we also tested whether ZIKV replication might be affected by small-molecule inhibitors of PD-L1 known as inhibitor 1 or BMS-1 [41] and inhibitor 3 [42]. Treatment of infected SF268 cells with either inhibitor did not suppress PD-L1 expression (S5E Fig) or viral load (S5F Fig), indicating that PD-L1 blockade *in vitro* does not suppress ZIKV infection. In other words, its effects *in vivo* are plausibly attributed to T cell response.

**Fig 4:**
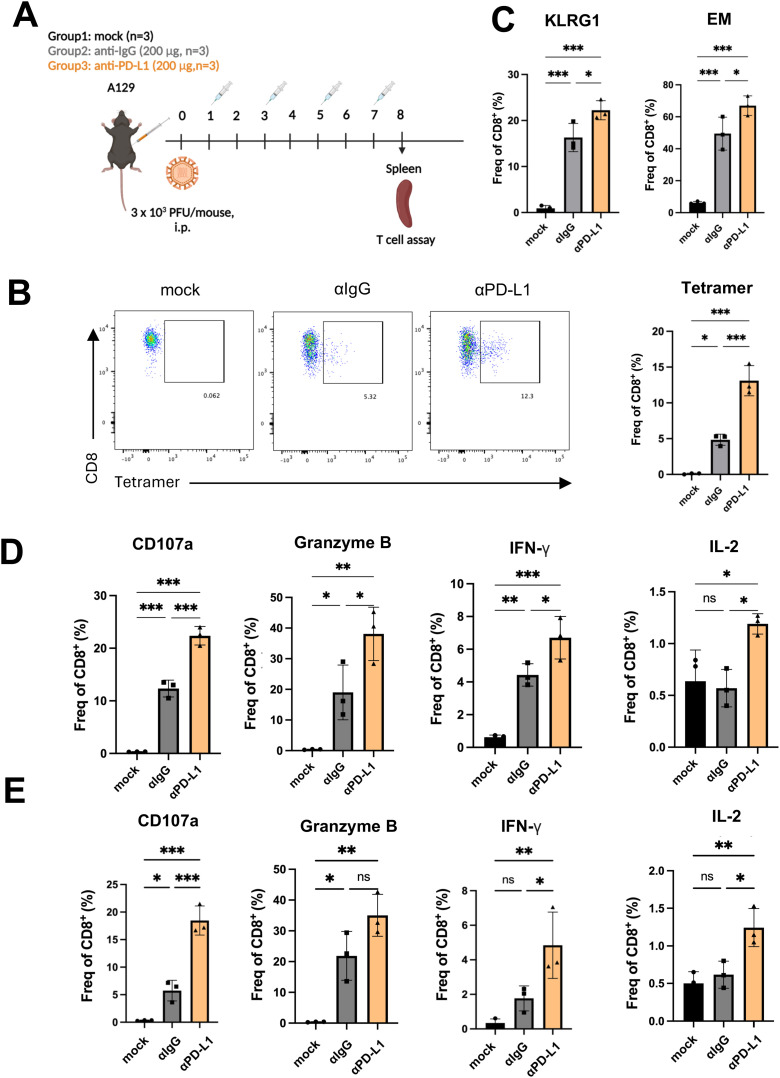
PD-L1 blockade elicits robust T cell response in mice. **(A)** Antibody treatment, viral challenge and sampling scheme for the A129 model. The figure was created using BioRender (https://BioRender.com). **(B)** Representative flow tracings and the number of tetramer-specific CD8^+^ T cells at 8 dpi. **(C)** Quantification of the frequency (Freq) of KLRG1^+^ CD8^+^ T cell and effector memory (EM) CD8^+^ T cells. Splenocytes were stimulated with 2 μg/ml E_4-12_ (**D**) and NS3_206-215_ (**E**) peptides for 5 hours in the presence of brefeldin **A.** The percentages of CD107a^+^ and cytokine-producing CD8^+^ T cells were assessed. The results are shown as the mean ± SD of three independent experiments. Statistical analyses were performed with one-way ANOVA (*P < 0.05; **P < 0.01; ***P < 0.001).

Tetramer assay was next used to assess antigen-specific T cell response (Fig 4A). We observed a more than 2-fold increase in E_4-12_ tetramer-positive CD8^+^ T cells in the splenocytes of the anti-PD-L1-treated group compared to the IgG-treated group on 8 dpi (Fig 4B). The frequencies of KLRG1^+^ CD8^+^ effector T cells and effector memory T cells were further induced upon anti-PD-L1 treatment (Fig 4C). The function of CD8^+^ T cells in combating virus-infected cells usually involves production of cytokines such as IL-2 and IFN-γ. To further assess T-cell functionality, splenocytes were stimulated with E_4-12_ (Fig 4D) and NS3_206–215_ (Fig 4E) peptides derived from viral proteins. The frequencies of CD8^+^ T cells expressing functional markers (CD107a and granzyme B) associated with cytotoxicity [43], as well as those producing IFN-γ and IL-2, consistently elevated in the PD-L1 antibody-treated mice following peptide stimulation (Fig 4D and 4E). These results indicate the potential of using anti-PD-L1 as an antiviral against ZIKV.

### NS4B induces PD-L1 expression by enhancing the activity of the PD-L1 promoter

**Fig 5:**
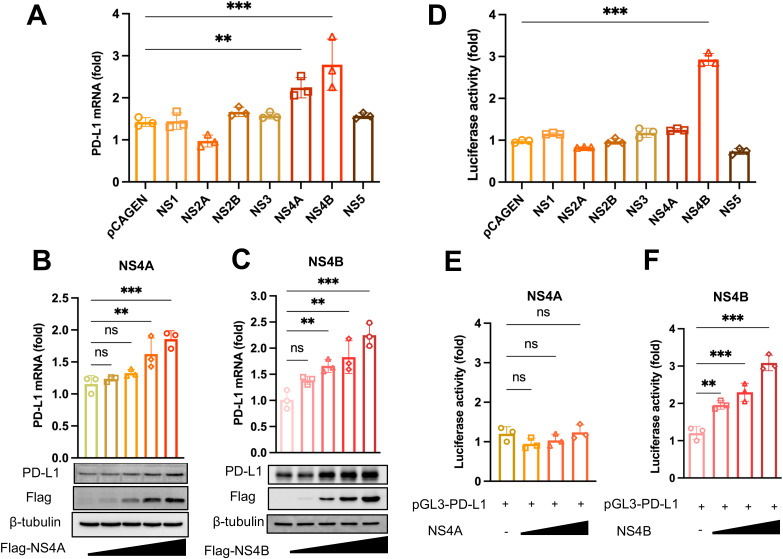
NS4B induces PD-L1 expression by activating the PD-L1 promoter. **(A)** SF268 cells were transfected with 2 μg of Flag-pCAGEN-NS1, NS2A, NS2B, NS3, NS4A, NS4B and NS5 plasmid, respectively. At 24 hours post-transfection, the mRNA levels of PD-L1 were assessed using RT-qPCR. GAPDH was used as a housekeeping control. SF268 cells were transfected with increasing dose of Flag-tag NS4A (**B**) and NS4B (**C**) expression plasmids. Western blotting was performed with β-tubulin as a loading control. The Western blotting results are representative of three independent experiments. **(D)** HEK293T cells were co-transfected with the NS plasmid and the PD-L1 promoter-driven reporter construct. At 24 hours post-transfection, luciferase activity was assessed using the Dual-Luciferase Reporter Assay System kit. HEK293T cells were co-transfected with increasing dose of NS4A (**E**) or NS4B (**F**) plasmid. The results are shown as the mean ± SD of three independent experiments. Statistical analyses were performed with one-way ANOVA (*P < 0.05; **P < 0.01; ***P < 0.001).

As ZIKV infection leads to increased PD-L1 expression in cultured cells and *in vivo*, we attempted to identify the specific viral proteins responsible for this induction. Through transient expression of individual ZIKV NS genes in SF268 cells, we observed that the expression of either NS4A or NS4B resulted in elevated PD-L1 mRNA levels (Fig 5A). Furthermore, both the mRNA and protein expression of PD-L1 showed a dose-dependent increase in response to NS4A and NS4B expression (Fig 5B and 5C). To further investigate the mechanism by which NS4A and NS4B induce PD-L1 expression, we examined their impact on the PD-L1 promoter activity and found a more than 2-fold activation of the PD-L1 promoter activity by NS4B (Fig 5D). A dose-dependent activation of the PD-L1 promoter activity was only seen with NS4B, not with NS4A (Fig 5E and 5F), suggesting that NS4B is the major viral protein that induces PD-L1 expression by enhancing the activity of the PD-L1 promoter.

PD-L1 is known to be upregulated by type I and type II IFN signaling [44]. In this regard, we have previously shown selective activation of IFN-γ signaling by ZIKV and its NS5 protein [19]. Although NS5 was not identified as an inducer of PD-L1 (Fig 5A and 5D), it would still be of interest to determine whether ZIKV might induce PD-L1 expression through IFN signaling. To address this, JAK-STAT inhibitor AG490 [19] was employed. Treatment of cells with AG490 reduced both basal and ZIKV-induced PD-L1 expression (S6A Fig). However, it remains to be further elucidated as to whether this effect might be ascribed to AG490-mediated suppression of ZIKV replication [19]. In another experiment, knockdown of STAT1 decreased basal PD-L1 expression but only minimally affected ZIKV-induced PD-L1 production (S6B Fig). In addition, depletion of neither STAT1 (S6C Fig) nor JAK1 (S6D Fig) altered NS4B-induced PD-L1 expression. Thus, whereas JAK/STAT signaling modulates basal expression of PD-L1, it contributes minimally to ZIKV-induced PD-L1 production. These results lent further support to our model of the role of PD-L1 during ZIKV infection *in vivo*.

### GRP78 interacts with NS4B to induce PD-L1 expression

**Fig 6:**
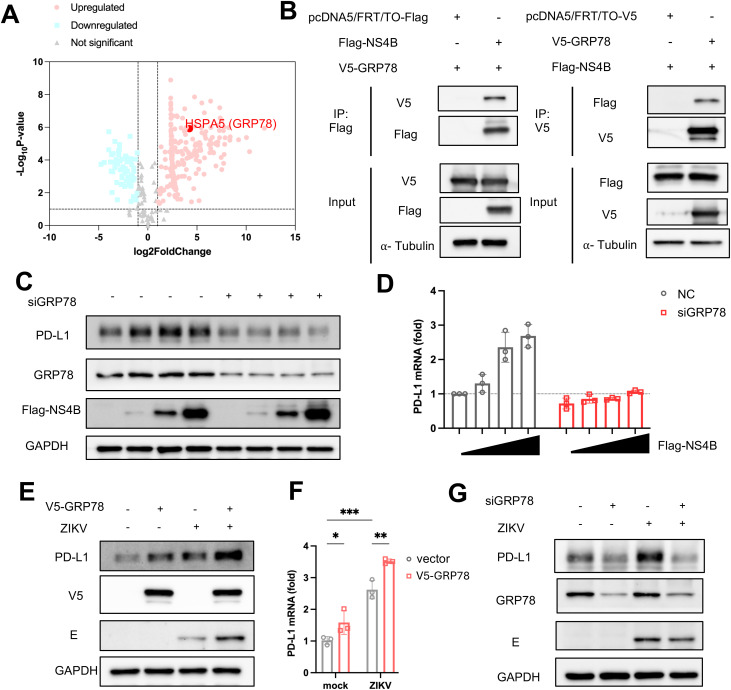
GRP78 interacts with NS4B to induce PD-L1 expression. **(A)** Volcano plot analysis of potential binding partners of NS4B. HSPA5 (GRP78) is highlighted with a red point. **(B)** Interaction between NS4B and GRP78. Immunoblot analysis of immunoprecipitates from HEK293T cells co-transfected with Flag-tagged NS4B and V5-tagged GRP78 expression plasmids. ⍺-Tubulin was used as a loading control. **(C)** Western blot analysis of PD-L1, GRP78, Flag-NS4B and GAPDH expression levels in control or GRP78-silenced SF268 cells. (**D**) mRNA levels of PD-L1 were assessed using RT-qPCR. GAPDH was used as a housekeeping control. Western blot (**E**) and RT-qPCR (**F**) analysis of PD-L1 and ZIKV E expression in SF268 cells transfected with V5-GRP78 expression plasmids for 24 hours, followed by ZIKV infection for 48 hours. **(G)** Western blot analysis of PD-L1, GRP78 and ZIKV E protein in SF268 cells transfected with control or GRP78 siRNAs for 24 hours, followed by ZIKV infection for 48 hours. Data are presented as the mean ± SD of three independent experiments. Statistical analyses were performed using Student’s t-test (*P < 0.05; ***P < 0.001).

To identify the host factors involved in NS4B-induced PD-L1 upregulation, we conducted mass spectrometric analysis to identify potential binding partners of NS4B. As shown in Fig 6A, HSPA5, also known as glucose-regulated protein 78 (GRP78), an endoplasmic reticulum (ER) stress regulator, was identified as a binding partner of NS4B. In this regard, it is noteworthy that ZIKV infection triggers ER stress [45,46] and that GRP78 stabilizes PD-L1 in triple-negative breast cancer [47].

To validate the interaction between NS4B and GRP78, we performed reciprocal co-immunoprecipitation assays. GRP78 was detected in the NS4B precipitate, while NS4B was also found in the GRP78 precipitate (Fig 6B). To investigate the role of GRP78 in NS4B-induced PD-L1 upregulation, we knocked down endogenous GRP78 in SF268 cells using siRNAs. The depletion of GRP78 abolished the enhanced PD-L1 expression at both the protein (Fig 6C) and mRNA levels (Fig 6D) in the presence of NS4B. Furthermore, overexpression of GRP78 further augmented PD-L1 expression following ZIKV infection (Fig 6E and 6F). Consistent with this, knockdown of GRP78 led to a reduction in PD-L1 expression in ZIKV-infected cells (Fig 6G). In addition, we observed that ZIKV infection induced an increase in GRP78 expression in SF268 cells (S7A Fig). These findings were in line with the model in which GRP78 interacts NS4B (S7B Fig) to upregulate PD-L1 expression.

## Discussion

In this study, we provided evidence that ZIKV infection induces the expression of PD-L1 in cultured cells and *in vivo*. Blockade of PD-1/PD-L1 immune checkpoint using anti-PD-L1 antibody effectively restricted ZIKV infection, ascribed to robust virus-specific CD8^+^ T cell response.

Recent studies have highlighted the critical roles of the PD-1/PD-L1 pathway in acute viral infection and pathogenesis [32]. PD-1/PD-L1 expression increased in various immune cells upon infection with severe ever with thrombocytopenia syndrome virus (SFTSV). A novel anti-PD-1 nanobody named NbP45 effectively inhibited SFTSV infection in a humanized mouse model [48]. Moreover, PD-L1 expression also elevated in cells infected with SARS-CoV-2 Omicron variant. In AAV-hACE2 mice infected with SARS-CoV-2, early PD-L1 blockade reduced proinflammatory cytokine levels and alleviated SARS-CoV-2-induced lymphopenia [49]. Consistent with these and several other studies with similar findings [34,35], we demonstrated increased PD-L1 expression during ZIKV infection and the effective suppression of ZIKV infection using a PD-L1-blocking antibody. In addition, we identified a specific viral protein, NS4B, that upregulates PD-L1 expression, likely through an interaction with cellular chaperone GRP78.

The regulation of PD-L1 expression involves both transcriptional and post-translational mechanisms [50,51]. Various transcription factors and signaling pathways have been shown to modulate PD-L1 expression. IFNs are key regulators of PD-L1 expression. Both IFN-α/β and IFN-γ activate JAK-STAT signaling to drive PD-L1 transcription by IRF1 in various cell types, including immune cells and cancer cells [44]. Thus, PD-L1 is a classical IFN-stimulated gene (ISG). Several crucial regulators of PD-L1 transcription, such as STAT3 and NF-κB, are activated during viral infection [50,51]. Moreover, histone modifications like histone H3 lysine 4 trimethylation (H3K4me3) and acetylation in the PD-L1 promoter are also influential in transcriptional regulation of PD-L1 expression [52]. On the other hand, PD-L1 stability and subcellular localization are modulated by post-translational modifications, including N-linked glycosylation, phosphorylation, ubiquitination and deubiquitination [50,51]. In our previous study, we demonstrated that ZIKV NS5 protein enhances IFN-γ activation and stimulates cellular proteins that drive inflammation, the known regulators of PD-L1 expression [19]. However, in the current study, we did not observe any direct influence of NS5 on PD-L1 expression (Fig 4A and 4D). Further analyses using a JAK-STAT inhibitor and the knockdown of STAT1 or JAK1 suggested that IFN signaling does not play a critical role in PD-L1 upregulation by ZIKV (S6 Fig). We found that both NS4A and NS4B of ZIKV can induce the expression of PD-L1 mRNA. Notably, the functions of flaviviral NS4A and NS4B in viral replication, membrane remodeling, immune evasion and pathogenesis are closely related [53]. While the role of NS4B in upregulating PD-L1 at the transcriptional level is supported by its ability to enhance promoter activity likely in an IFN-independent manner (S6D Fig), the mechanism by which NS4A upregulates PD-L1 expression remains unclear and awaits further investigations.

We found that ZIKV might induce PD-L1 expression through the interaction between NS4B and GRP78. GRP78 belongs to the heat shock protein 70 (HSP70) family [54]. Primarily localized in the ER, GRP78 functions as a molecular chaperone, facilitating proper protein folding and preventing the accumulation of unfolded and misfolded proteins [55]. Under normal physiological conditions, GRP78 maintains proteostasis within the ER. Upon ER stress, its expression is upregulated to meet increased demand of protein folding [56]. Beyond the ER, GRP78 is also present on the cell surface as well as in the nucleus and mitochondria. It can also be secreted [57].

ZIKV infection is known to induce ER stress. This is evidenced by the increased levels of ER stress markers observed during infection including GRP78 as indicated in our study. Sustained ER stress in ZIKV-infected cells leads to apoptosis [45]. GRP78 has been implicated in the replication cycle of several flaviviruses, including dengue virus, Japanese encephalitis virus, and ZIKV [57]. Notably, GRP78 has been shown to stabilize PD-L1 in triple-negative breast cancer [47]. Interaction between GRP78 and other ZIKV proteins such as E and NS1 has also been reported [57]. In this study, we identified an interaction between GRP78 and ZIKV NS4B, a viral protein with diverse roles in viral replication and immune evasion [58]. Other interaction partners of NS4B such as VDAC2 and SLC25A1 identified by mass spectrometry are associated with cellular metabolic processes (Fig 6A). We focused on GRP78 due to its role as an ER stress marker and its impact on PD-L1 regulation. We demonstrated a proviral role of GRP78 in SF268 cells. This aligns with findings from SARS-CoV-2 infection, where GRP78 acts as a proviral factor [59]. Several studies have also shown that knockdown of GRP78 significantly reduces ZIKV production [60–62]. Our results suggest that GRP78 might serve as a critical link between ZIKV NS4B and PD-L1 upregulation. With the help of the AlphaFold program, we predicted the specific interaction domain and key amino acids in both NS4B and GRP78 (S7B Fig). Further studies are needed to elucidate the precise mechanism by which NS4B and GRP78 regulate PD-L1 transcription and protein stability. In this regard, characterization of NS4B-deficient ZIKV constructed with an infectious clone could be insightful. It will also be of interest to identify natural variants of NS4B that exhibit differential activity in the interaction with GRP78 and the activation of PD-L1 expression. Notably, an NS4B mutant of ZIKV is severely attenuated and potently induces antiviral immunity including CD8^+^ T cell response [63]. It will be intriguing to determine whether GRP78 and PD-L1 might play any role in this phenotype.

Several mouse models have been developed for the study of ZIKV infection and pathogenesis [64,65]. Most of these models involve mice that lack a fully functional immune system. For example, A129 mice displayed severe symptoms following subcutaneous challenge in the lower leg to simulate a mosquito bite [40]. AG129 mice that are deficient in both type I and type II IFN receptors can also be used. Whereas 3-week-old A129 and AG129 mice exhibited paralysis and succumbed by 7 dpi, older A129 mice of 11-week-old experienced weight loss and viremia but recovered after 8 dpi [66,67]. One major drawback of using immunocompromised mice is that they may not accurately reflect the natural immune response to ZIKV infection. Other studies have shown that ZIKV can infect WT C57BL/6 mice and trigger an antiviral T cell response, although resulting in only a mild decrease in body weight [23]. For our study, we used 6–8-week-old A129 mice as the ZIKV infection model. It was based on our *in vitro* findings indicating that STAT1 or STAT3 signaling was not associated with ZIKV-induced PD-L1 upregulation, as well as the necessity to assess the efficacy of PD-L1-blocking antibody treatment. While a deficiency in type I IFN could compromise CD8^+^ T cell response to viral infection [68], our results demonstrated a robust virus-specific CD8^+^ T cell response and improved functionality of CD8^+^ T cells upon treatment with an anti-PD-L1 antibody, validating the suitability of our mouse model. Additionally, our data showed increased production of ZIKV-specific memory CD8^+^ T cells in A129 mice treated with PD-1/PD-L1 antibodies. Collectively, these findings offer valuable insights into the development of a novel therapeutic approach against ZIKV infection.

Although the PD-L1 antibody-treated group showed a higher survival rate, the difference was not statistically significant. The IFN-α/β receptor deficiency in A129 mice confers extreme susceptibility to ZIKV infection in a lethal infection model [64,65]. The absence of type I IFN signaling enables uncontrolled viral replication, resulting in rapid disease progression that might render the comparison of survival differences between control and anti-PD-L1 treatment groups more difficult. In our study, we observed significant differences in clinical scores and viral loads between the two groups, despite similar mortality. Further optimization of the experimental settings including antibody dosage, virus strain and administration route, as in the case of *Ifnar*^-/-^ mice [69,70], might allow us to demonstrate the beneficial effect of PD-L1 blockade more convincingly.

Our demonstration of the activation of PD-L1 immune checkpoint by ZIKV raises several interesting questions that merit further investigations. First, it will be of interest to see whether the activation of PD-L1 immune checkpoint could be induced by other flaviviruses. If our findings with ZIKV can be generalized to other human flaviviral pathogens and other flaviviral NS4B proteins, they should have implications in the development of antivirals and vaccines. Second, the relevance of PD-L1 immune checkpoint to re-exposure should be addressed. CD8^+^ T cell response is critical not only to ZIKV-naïve individuals, but also to people who have previously be exposed to or vaccinated against ZIKV and closely related flaviviruses such as Dengue virus, Japanese encephalitis virus and yellow fever virus. Cross-protective T cell response against these viruses has just begun to be understood [71–73]. Further analysis of the role of PD-L1 checkpoint in cross protection and re-exposure is warranted. Third, whether and how the PD-L1 checkpoint might affect vaccination and vaccine protection are related questions that require further analysis. The importance of CD8^+^ T cell response in vaccine protection against flaviviruses has been documented [74,75]. Clarification of the role of PD-L1 checkpoint in this context might lead to new strategies in vaccine design. For example, whether immune checkpoint blockade might be of any benefit in vaccination should be determined.

One limitation of our study is the absence of clinical samples from ZIKV-infected individuals to assess PD-L1 expression. This is primarily due to the scarcity of imported cases of human ZIKV infection in our region. Furthermore, ascribed to the lack of humanized mouse models for ZIKV infection [64,65], our study did not include clinically approved PD-1/PD-L1 antibodies such as Nivolumab, Pembrolizumab and Tislelizumab. CD8^+^ T cell response is cytotoxic by definition and could trigger pathogenic inflammation and tissue damage in the context of ZIKV infection [4,5]. Further investigations are required to explore the clinical significance and safety profile of immune checkpoint therapy targeting PD-1/PD-L1 during ZIKV infection. Particularly, since CD8^+^ T cell response has been linked to ZIKV pathogenesis in the brain [26] and other immune-privileged organs [76], it will be of importance to verify that immune checkpoint blockade is free of severe side effects before any tests can be started in humans.

## Methods

### Ethics statement

The animal experiments were approved by the Committee on the Use of Live Animals in Teaching and Research of the University of Hong Kong (approval number: CULATR 24–087) and performed according to established safety protocols in the biosafety level 2 laboratory at the University of Hong Kong.

### Viruses, plasmids and cells

ZIKV Puerto Rico strain PRVABC59 was obtained from the ATCC (VR-1843). For virus propagation, C6/36 cells grown for 24 hours were infected with ZIKV and maintained in DMEM with 2% FBS at 28°C in a 5% CO_2_ atmosphere. Virus was harvested on day 4 after infection and spun at 2,000 × g for 10 minutes to remove cell debris. The viral supernatant was aliquoted and frozen at -80°C.

NS genes (NS1, NS2A, NS2B, NS3, NS4A, NS4B, and NS5) of ZIKV were PCR amplified from PRVABC5 cDNA and cloned into the pcDNA5/FRT/TO or pCAGEN vector. pcDNA-GRP78-V5 were constructed as previously described [77].

All cells were grown in the presence of 10% fetal calf serum (FCS) and 1% penicillin-streptomycin in a 5% CO_2_ atmosphere. HEK293T, VeroE6 and SF268 cells were in Dulbecco’s modified Eagle’s medium at 37°C. C6/36 cells were in the same medium at 28°C. JEG3 cells were in minimum essential medium at 37°C. Cells were transfected with Lipofectamine 3000 Transfection Reagent (ThermoFisher) or jetPRIME (Polyplus). The peripheral blood mononuclear cells (PBMCs) were isolated as described previously [78]. GM-CSF and IL-4 were used to induce dendritic cell differentiation.

### Antibodies

The following antibodies were used for Western blotting: mouse monoclonal anti-Flag antibody M2 (Sigma, F1804), flavivirus group antigen antibody (Novus Biologicals, NBP2–52709), recombinant rabbit monoclonal anti-PD-L1 antibody (Abcam, ab205921), rabbit anti-GRP78 (Abcam, ab21685), mouse monoclonal anti-α-tubulin antibody (Sigma, T5168), anti-GAPDH antibody (Santa Cruz, sc-47724) and anti-β-actin antibody (Santa Cruz, sc-8432). A recombinant mouse monoclonal anti-PD-L1 antibody, designated anti-PD-L1-mIgG1e3 or Azeto (InvivoGen, pdl1-mab15–10), was employed for PD-L1 blockade in mice.

### Viral infection

SF268 or JEG3 cells were infected with ZIKV at an MOI of 1.0 or 0.1. Following a 1-hour incubation at 37°C, the cells were washed at least twice with PBS and then incubated in DMEM containing 2% FCS. Cell lysates or supernatants were collected at the specified time points post-infection. Three independent experiments were performed.

### Plaque assay

Confluent VeroE6 cells were seeded in a 12-well plate and incubated with 10-fold serial dilutions of ZIKV for 1 hour. After adsorption to VeroE6 cells, the viral supernatant was removed, and the cells were washed three times with PBS before being overlaid with 1% low melting point agarose in DMEM. The cells were then incubated at 37°C for 4 days, fixed with 10% formaldehyde overnight, and subsequently stained with 1% crystal violet to visualize and count the plaques for determining viral titers.

### Western blotting and co-immunoprecipitation

Western blotting was performed as described previously [79]. In brief, cells were directly lysed in lysis buffer (50 mM Tris-Cl at pH 7.4, 150 mM NaCl, 1% NP-40, 5 mM EDTA, 1 × complete protease inhibitor cocktail, and 10% glycerol) which mixed with SDS sample buffer. Cells were sonicated and boiled. The protein samples were resolved by SDS-PAGE and then transferred onto a polyvinylidene difluoride membrane. The membranes were blocked with 5% skimmed milk in TBST (TBS containing 0.5% Tween 20) for 1 hour at room temperature and then incubated with primary antibodies overnight at 4°C, followed by an incubation with secondary antibodies for 1 hour at room temperature. The reactive protein bands were visualized using an enhanced chemiluminescence reagent (ThermoFisher, 34580) with a ChemiDoc Imaging System (Bio-Rad). Co-immunoprecipitation was performed as described previously [80]. In brief, cells were lysed in lysis buffer and cell lysates were incubated with anti-Flag M2 beads (Sigma-Aldrich) for 2–4 hours at 4˚C. The beads were then washed three times and boiled. Protein samples were analyzed by Western blotting.

### RNA extraction and quantitative reverse transcription PCR (RT–qPCR)

RNA extraction, reverse transcription, and qPCR were performed as previously described [81]. Briefly, total RNA was extracted from cells or mouse organs (brain, testis, and spleen) with a RNeasy Mini Kit (Qiagen, Germantown, MD, USA) and reverse-transcribed with a PrimeScript RT reagent Kit with gDNA Eraser (Perfect Real Time, Takara, R0047) or One-Step TB Green PrimeScript RT-PCR Kit (Perfect Real Time, Takara, R0086). Real-time PCR was performed using ChamQ SYBR Color qPCR Master Mix (Vazyme, Q411-02) and a CFX96 Touch Deep Well Real-Time PCR Detection System (Bio–Rad, Berkeley, California, USA) according to the manufacturer’s instructions. The primers used were listed below: 5′-TGGCATTTG CTGAACGCAT TT and 5′-TGCAGCCAGG TCTAATTGTT TT for human PD-L1; 5′- AGAAGGCTGG GGCTCATT TG and 5′-CTGTGGTCAT GAGTCCTTC for human GAPDH; 5′-CATGGCTTCT GACAGCCGCT and 5′-TGGATGCTCT TCCCGGTCAT TTT for ZIKV E as well as 5′- AGGTCGGTGT GAACGGATTT G and 5′-GGGGTCGTTG ATGGCAACA for mouse GAPDH. Relative gene expression was determined either through normalization to the corresponding Ct values for GAPDH and calculated using the 2^-ΔΔCt^ method.

### Evaluation of the effect of PD-L1 blockade on ZIKV infection *in vivo*

The type I IFN receptor-deficient A129 mouse model was utilized for ZIKV infection as previously described [46,82,83]. In brief, 6–8 week-old A129 male mice were randomly split into three groups. The mice were inoculated i.p. with 3 × 10^3^ PFU of ZIKV at a final volume of 100 μL. The mice were i.p. treated with 200 μg isotype control (anti-β-Gal-mIgG1e3, InvivoGen) or anti-PD-L1 antibody (anti-PD-L1-mIgG1e3 or Azeto, InvivoGen, pdl1-mab15–10) at a final volume of 100 μL on days 1, 3, 5 and 7 post-infection. Mock treatment was used as control. The mice were euthanized on day 8 post-virus challenge for sample harvest. Alternatively, the mice were euthanized for humane end points when there was a > 20% weight loss. During sample harvest, the brain and testis of mice were collected for viral load studies, and the spleen of mice were also used for splenocyte isolation. Mice were monitored each day for clinical signs and a numerical score was assigned at each observation as previously described (0, normal; 2, ruffled fur; 3, lethargy, pinched, hunched, wasp waisted; 5, laboured breathing, rapid breathing, inactive, neurological; and 10, immobile) [40]. Body weight and survival were monitored daily for 14 dpi or until euthanasia.

### Flow cytometry

Splenocytes were isolated and filtered through a 70-μm cell strainer (Falcon) and then resuspended in RC-10 medium (RPMI 1640 medium supplemented with 10% FCS, 2mM L-glutamine, 100 U/ml penicillin, 100 mg/ml streptomycin, 50 mM 2-mercaptoethanol and 10mM HEPES). Red blood cells were lysed using Red Blood Cell Lysing Buffer (Sigma, R7757). Cells were treated with the 2.4G2 monoclonal antibody (anti-CD16/32) to block Fc receptors. To exclude dead cells from the staining results, Zombie Aqua Fixable Viability Kit (Biolegend, 423102) was used according to the manufacturer’s instructions. The following antibodies against mouse antigens were used: FC block (BioLegend 156604), anti-CD4 (PerCP-Cy5.5, BioLegend 116012), anti-CD8 (BV421, BioLegend 100753), anti-CD44 (BV711, BioLegend 103057), anti-KLRG1 (APC, BioLegend 138412), anti-CD62L (PE-Cy7, BioLegend 104418), anti-PD-1 (PE, BioLegend 135205), anti-CD107a (A488, BioLegend 121608), anti-IFN-γ (PE-Cy7, BioLegend 505825), anti-TNF-α (BV785, BioLegend 506341), anti-IL-2 (APC, BioLegend 503810) and anti-granzyme B (PE, BioLegend 396406). Antigen-specific CD8^+^ T cells were detected using ZIKV-specific textramers (Immudex). For intracellular cytokine staining, a total of 1 × 10^6^ splenocytes were plated in each well of 96-well U-bottom plates and stimulated with individual peptide (E_4-12_: IGVSNRDFV and NS3_206–215_: APTRVVAAEM). Peptides were synthesized at GL Biochem (Shanghai, China) with 95% purity. Splenocytes stimulated with PMA-ionomycin were used as a positive control. After 1 hour, brefeldin A (GolgiPlug, BD Biosciences) was added to the cells, and the cells were incubated for 5 hours at 37°C. A BD Cytofix/Cytoperm Plus Fixation/Permeabilization Kit (BD Bioscience) was then used according to the manufacturer’s instructions. All samples were acquired on an LSR Fortessa flow cytometer (BD Biosciences, USA), and the data were analyzed with FlowJo software.

### Luciferase reporter activity of PD‐L1 promoter

Genome DNA was extracted using the Quick-DNA Miniprep Plus Kit (Zymo Research). The PD-L1 promoter was generated through PCR amplification. The resulting PCR product was purified with the FastPure Gel DNA Extraction Mini Kit (Vazyme) and then inserted into the pGL3-basic firefly luciferase vector. In the reporter assays, HEK293T cells were co-transfected with the ZIKV NS gene, the PD-L1 promoter, and the Renilla construct using the GeneJuice Transfection Reagent (Sigma). At 24 hours post-transfection, luciferase activity was assessed using the Dual-Luciferase Reporter Assay System kit (Promega).

### RNA interference

siRNAs against GRP78 were transfected using Lipofectamine 3000 (Invitrogen) at a final concentration of 75 pmol for 2 consecutive days in 6-well plates following the manufacturer’s instructions. siRNAs against GRP78 (5′- UCUACAGCUU CUGAUAAUCA ACCAA) are constructed by Integrated DNA Technologies.

### Proteomics

To identify NS4B-binding proteins, NS4B and its binding proteins were precipitated with Anti-Flag M2 agarose beads from HEK293T cells stably expressing Flag-NS4B. After washing five times with 20 mM ammonium bicarbonate (pH:7.8), 10 mM DTT in 100 mM ammonium bicarbonate (pH:7.8) was added to the beads for reduction in a 37°C shaker for 30 min. Then, alkylation was performed by adding an equal volume of 25 mM iodoacetamide to the DTT bead suspension and rotating in the dark for 30 minutes at room temperature. Proteins were then digested by trypsin (1:100 enzyme/substrate ratio, 37°C shaker, overnight). Peptides were desalted with C18 Toptip and then dried in a rotary evaporator.

Purified peptides were re-suspended in 0.1% formic acid for LC-MS run. Briefly, peptides separated with the C18 Acclaim PepMap column (75 μm id × 15 cm, 2 μm particle sizes, 100 Å pore sizes, Thermo Scientific) were ionized at 1.9 kV in the positive ion mode. MS1 survey scans were acquired at the resolution of 70,000 from 350 to 1800 m/z, with a maximum injection time of 100 ms and automated gain control (AGC) target of 1e6. MS/MS fragmentation of the 14 most abundant ions were analyzed at a resolution of 17,500, AGC target 5e4, maximum injection time 65 ms, and normalized collision energy of 26. Dynamic exclusion was set to 20 s and ions with a charge of +1, + 7 and>+7 was excluded. MS/MS fragmentation spectra were searched with Proteome Discoverer SEQUEST (version 2.4, Thermo Scientific) against the in silico tryptic digested Uniprot all-reviewed Homo sapiens database (release June 2017, 42,140 entries). The maximum missed cleavages were set to 3. Dynamic modifications were set to oxidation on methionine (M, + 15.995 Da) and deamidation on asparagine and glutamine (N and Q, + 0.984 Da). Carbamidomethylation on cysteine residues (C, + 57.021 Da) was set as a fixed modification. The maximum parental mass error was set to 10 ppm, and the MS/MS mass tolerance was set to 0.03 Da. The false discovery threshold was set strictly to 0.01 using the Percolator Node validated by q-value. The relative abundance of parental peptides was calculated by integration of the area under the curve of the MS1 peaks using the Minora LFQ node. Spectral annotation was generated by the Interactive Peptide Spectral Annotator (IPSA, http://www. interactivepeptidespectralannotator.com/). The mass spectrometry data have been deposited into the ProteomeXchange Consortium via the PRIDE partner repository with the dataset identifier PXD062250 (https://www.ebi.ac.uk/pride/archive/projects/ PXD062250).

### RNA-seq data analysis

RNA-seq data from JEG3, U-251 MG and HK-2 cells infected with ZIKV were retrieved from NCBI Gene Expression Omnibus [38]. DEGs were detected by two-tailed Student’s t-test by comparing with corresponding mock infection. In each group, those genes with P values less than 0.05 and FC larger than 1.5 or less than 0.5 were detected as DEGs. DEGs were enriched through Gene Ontology Biological Process (GO BP) .

## Supporting information

S1 Fig RNA-seq data for JEG3, U-251 MG and HK-2 cells infected with ZIKV. (**A**) DEGs in placental cells (JEG-3), nerve cells (U-251 MG) and kidney cells (HK-2) retrieved from the publicly available datasets. In each group, those genes with P values less than 0.05 and fold change larger than 1.5 or less than 0.5 were detected as DEGs. (**B**) Gene Ontology Biological Processes (GO BP) analysis of the 92 common DEGs. (**C**) A heatmap depicting the expression of 92 common DEGs across JEG-3, U-251 MG and HK-2 cells. Rows represent genes, and columns represent samples obtained from the three cell lines: JEG-3 cells, U-251 MG cells and HK-2 cells. Each cell line includes both non-infected and ZIKV-infected groups, with three experiments conducted for each sample. The numerical values in the heatmap represent the logarithm of the expression value + 1.
(TIF)

S2 Fig ZIKV infection upregulates PD-L1 expression in JEG3 cells. Flow cytometric analysis of PD‐L1 in JEG3 cells upon ZIKV infection. JEG3 cells were infected with ZIKV strain PRVABC59 for 48 hours and were analyzed for the expression of PD-L1 (gated on live cells) (**A**), quantification of the frequency of PD-L1 positive cells (**B**), quantification of the mean fluorescence intensity (MFI) of PD-L1 using flow cytometry (**C**). The results are shown as the mean ± SD of three independent experiments. Statistical analyses were performed with one-way ANOVA (*P < 0.05; **P < 0.01; ***P < 0.001).
(TIF)

S3 Fig ZIKV Uganda strain upregulates PD-L1 expression in cultured cells. SF268 cells (**A**) and JEG3 cells (**B**) were infected with ZIKV AF-976 Uganda strain at a MOI of 1. Cell lysates were collected at 24, 48 and 72 hpi. The mRNA levels of PD-L1 were assessed using RT-qPCR. GAPDH was used as a housekeeping control. Data are presented as the mean ± SD of three independent experiments. Statistical analyses were performed by two-way ANOVA (***P < 0.001). Protein levels of PD-L1 and ZIKV E were assessed by Western blotting with GAPDH as a loading control.
(TIF)

S4 Fig Impact of PD-L1 blockade on clinical signs and survival of A129 mice. (**A**) Antibody treatment and viral challenge scheme for the A129 model for survival. Male A129 mice (n = 8/group) were i.p. inoculated with 2 × 10³ PFU of ZIKV and subsequently i.p. treated with 150 μg of either IgG control or anti-PD-L1 antibody on 2, 4, 6, and 8 dpi. Body weight changes in mice over a 14-day period were observed. The figure was created using BioRender (https://BioRender.com). (**B**) Clinical scores of A129 mice. The results are shown as the mean ± SD of three independent experiments. Statistical analyses were performed with two-way ANOVA (*P < 0.05; **P < 0.01; ***P < 0.001). (**C**) Survival rate of mice. Mice that lost more than 20% of their basal weight were euthanized.
(TIF)

S5 Fig PD-L1 inhibition does not significantly affect ZIKV infection *in vitro.* (**A**) U251 cells were transfected with control or PD-L1 siRNA for 24 hours, followed by ZIKV infection for 48 hours. Protein levels of PD-L1 and E protein were assessed by Western blotting with GAPDH as a loading control. (**B**) ZIKV RNA levels in the supernatant of infected cells were detected by one-step RT-qPCR. Statistical analyses were performed with one-way ANOVA. (**C**) SF268 cells were transfected with increasing dose of PD-L1. Protein levels of PD-L1 and E protein were assessed by Western blotting with ⍺-tubulin as a loading control. Results are representative of three independent experiments. (**D**) SF268 and U251 cells were infected with ZIKV at an MOI of 1. After 1 hour, the medium was replaced with 2% DMEM containing either IgG control (αIgG) or anti-PD-L1 (αPD-L1; Azeto) at 2 µg/ml. Viral RNA levels in the supernatant were quantified by one-step RT-qPCR at 24 hpi. Statistical significance was determined using Student’s t-test. **(E)** SF268 cells were infected with ZIKV at an MOI of 1. After a 1-hour incubation, the medium was replaced with 2% DMEM containing either PD-1/PD-L1 inhibitor 1 (BMS-1, 1 μM) or PD-1/PD-L1 inhibitor 3 (2 μM). Cell lysates were collected at 48 hpi. The mRNA levels of PD-L1 were assessed using RT-qPCR. GAPDH was used as a housekeeping control. Data are presented as the mean ± SD of three independent experiments. Statistical analyses were performed by two-way ANOVA. **(F)** ZIKV RNA levels in the supernatant of infected cells were detected by one-step RT-qPCR. Statistical analyses were performed with one-way ANOVA.
(TIF)

S6 Fig Influence of JAK/STAT inhibition on ZIKV-induced PD-L1 expression *in vitro.* (**A**) Effect of AG490 on ZIKV-induced PD-L1 expression. SF268 cells were infected with ZIKV at an MOI of 1 for 24 hours and then treated with 50 μM AG490 for 12 hours. Western blot analysis of PD-L1, STAT3, pSTAT3 and ZIKV E protein in SF268 cells was performed with GAPDH as a loading control. Effect of STAT1 knockdown on PD-L1 expression induced by ZIKV (**B**) and NS4B (**C**). Western blot analysis of PD-L1, STAT1, ZIKV E/Flag-NS4B and GAPDH/α-tubulin expression levels in control or STAT1-silenced SF268 cells was performed. (**D**) Effect of JAK1 knockdown on NS4B-induced PD-L1 expression. Western blot analysis of PD-L1, JAK1, Flag-NS4B and α-tubulin expression levels in control or JAK1-silenced SF268 cells was performed.
(TIF)

S7 Fig GRP78 induces PD-L1 expression during ZIKV infection. (**A**) Western blot analysis of PD-L1, GRP78 and ZIKV E protein in SF268 cells after ZIKV infection for 24, 48 and 72 hours. (**B**) AlphaFold analysis of the specific interaction domain and key amino acid site in both NS4B and GRP78. Blue: NS4B, pink: GRP78.
(TIF)
