# Supplementary figures and images for: Activation of PD-1/PD-L1 immune checkpoint by Zika virus
Source: PLoS Pathog. 2025 Sep 8;21(9):e1013457. doi: 10.1371/journal.ppat.1013457 (PMC12431654; doi:10.1371/journal.ppat.1013457)

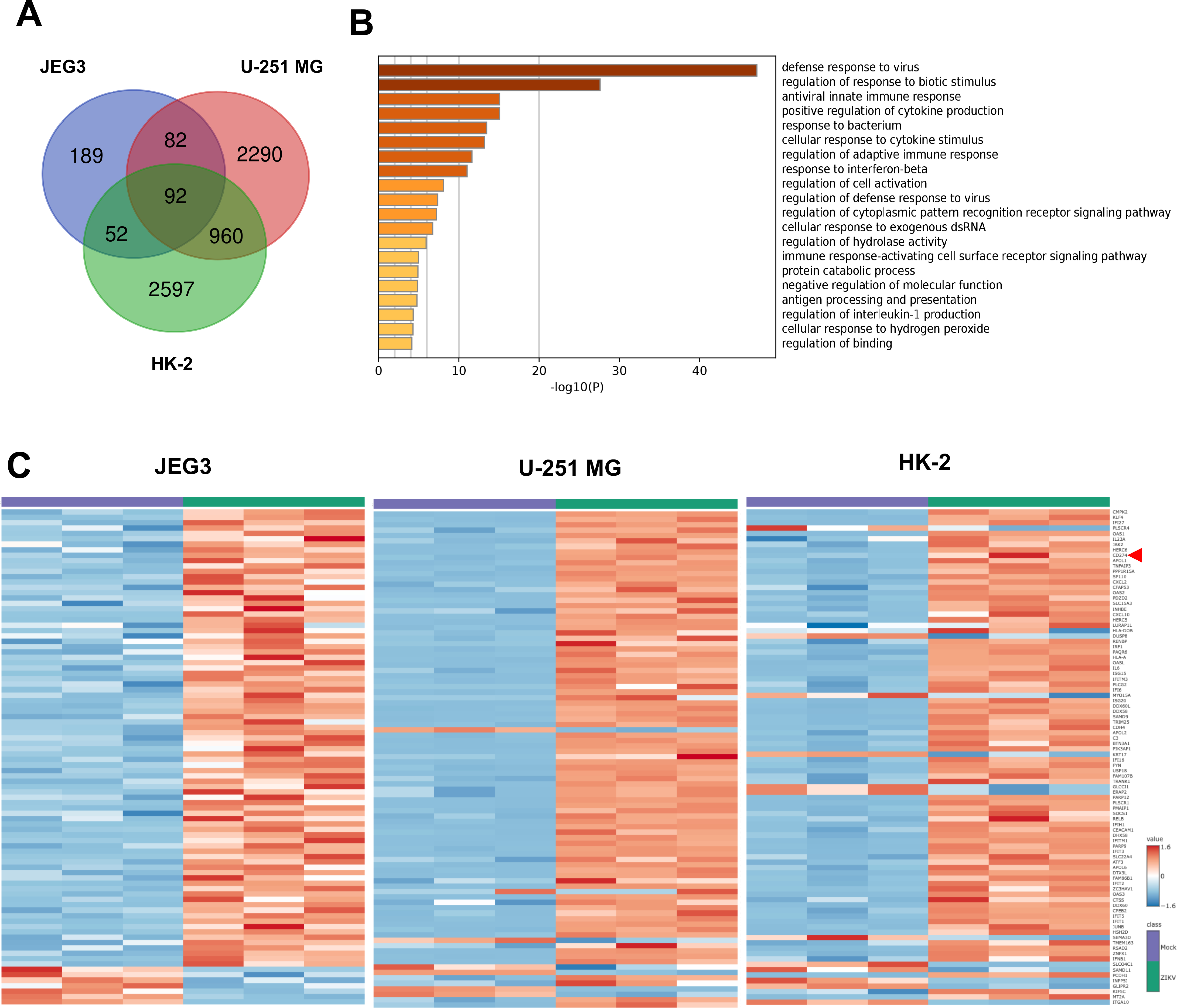

Supplement: S1 Fig — (A) DEGs in placental cells (JEG-3), nerve cells (U-251 MG) and kidney cells (HK-2) retrieved from the publicly available datasets. In each group, those genes with P values less than 0.05 and fold change larger than 1.5 or less than 0.5 were detected as DEGs. (B) Gene Ontology Biological Processes (GO BP) analysis of the 92 common DEGs. (C) A heatmap depicting the expression of 92 common DEGs across JEG-3, U-251 MG and HK-2 cells. Rows represent genes, and columns represent samples obtained from the three cell lines: JEG-3 cells, U-251 MG cells and HK-2 cells. Each cell line includes both non-infected and ZIKV-infected groups, with three experiments conducted for each sample. The numerical values in the heatmap represent the logarithm of the expression value + 1. (TIF) [file ppat.1013457.s001.tif]

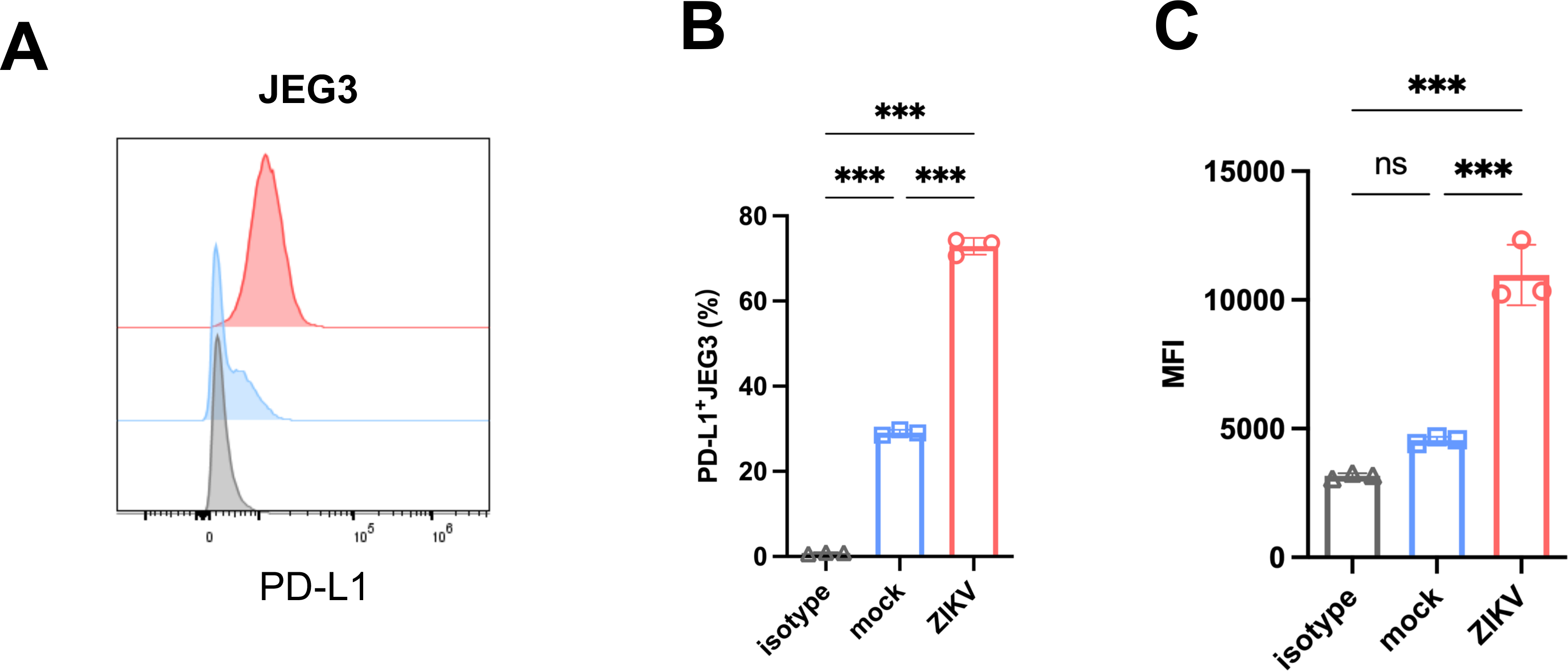

Supplement: S2 Fig — Flow cytometric analysis of PD‐L1 in JEG3 cells upon ZIKV infection. JEG3 cells were infected with ZIKV strain PRVABC59 for 48 hours and were analyzed for the expression of PD-L1 (gated on live cells) (A), quantification of the frequency of PD-L1 positive cells (B), quantification of the mean fluorescence intensity (MFI) of PD-L1 using flow cytometry (C). The results are shown as the mean ± SD of three independent experiments. Statistical analyses were performed with one-way ANOVA (*P < 0.05; **P < 0.01; ***P < 0.001). (TIF) [file ppat.1013457.s002.tif]

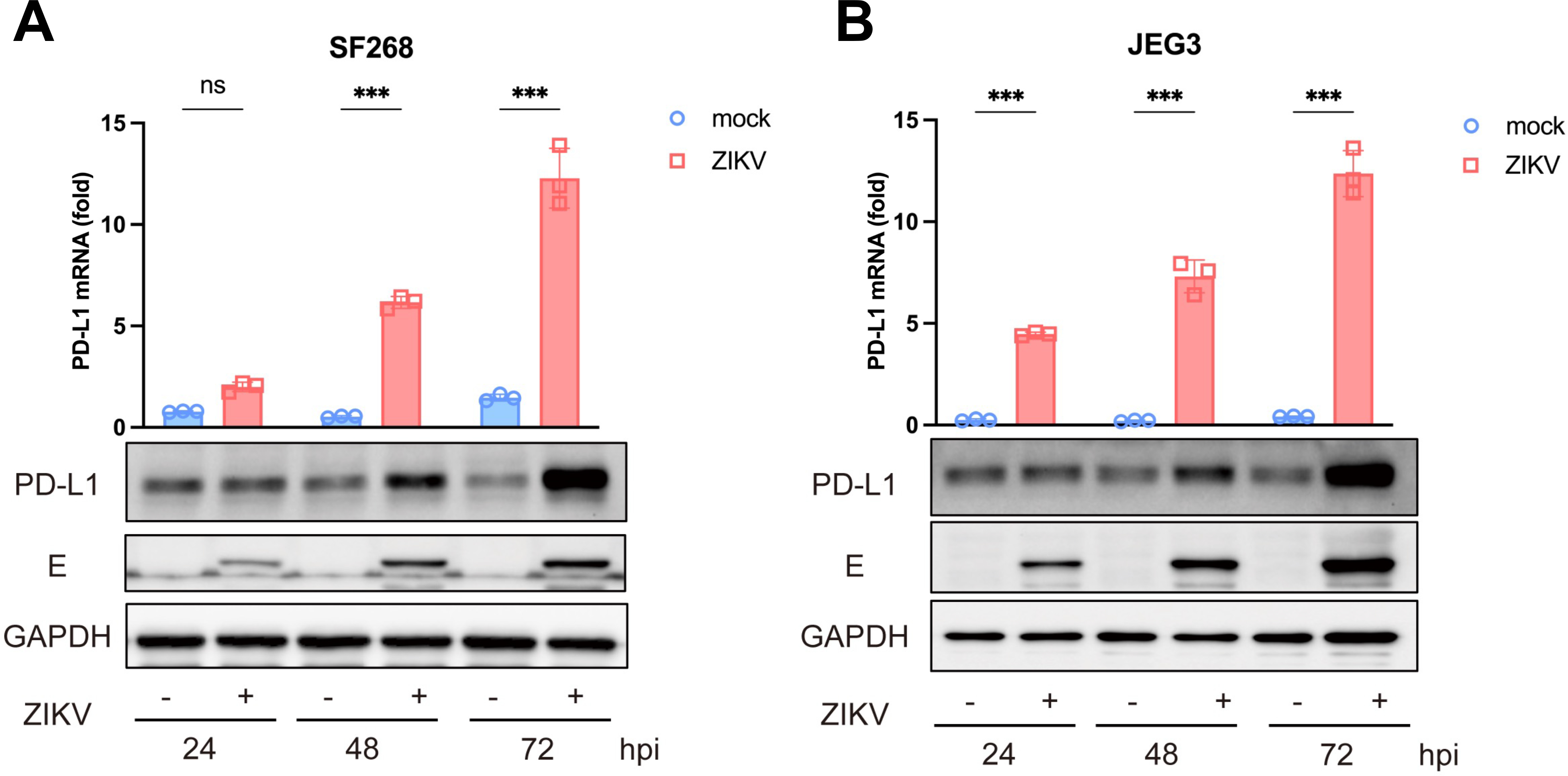

Supplement: S3 Fig — SF268 cells (A) and JEG3 cells (B) were infected with ZIKV AF-976 Uganda strain at a MOI of 1. Cell lysates were collected at 24, 48 and 72 hpi. The mRNA levels of PD-L1 were assessed using RT-qPCR. GAPDH was used as a housekeeping control. Data are presented as the mean ± SD of three independent experiments. Statistical analyses were performed by two-way ANOVA (***P < 0.001). Protein levels of PD-L1 and ZIKV E were assessed by Western blotting with GAPDH as a loading control. (TIF) [file ppat.1013457.s003.tif]

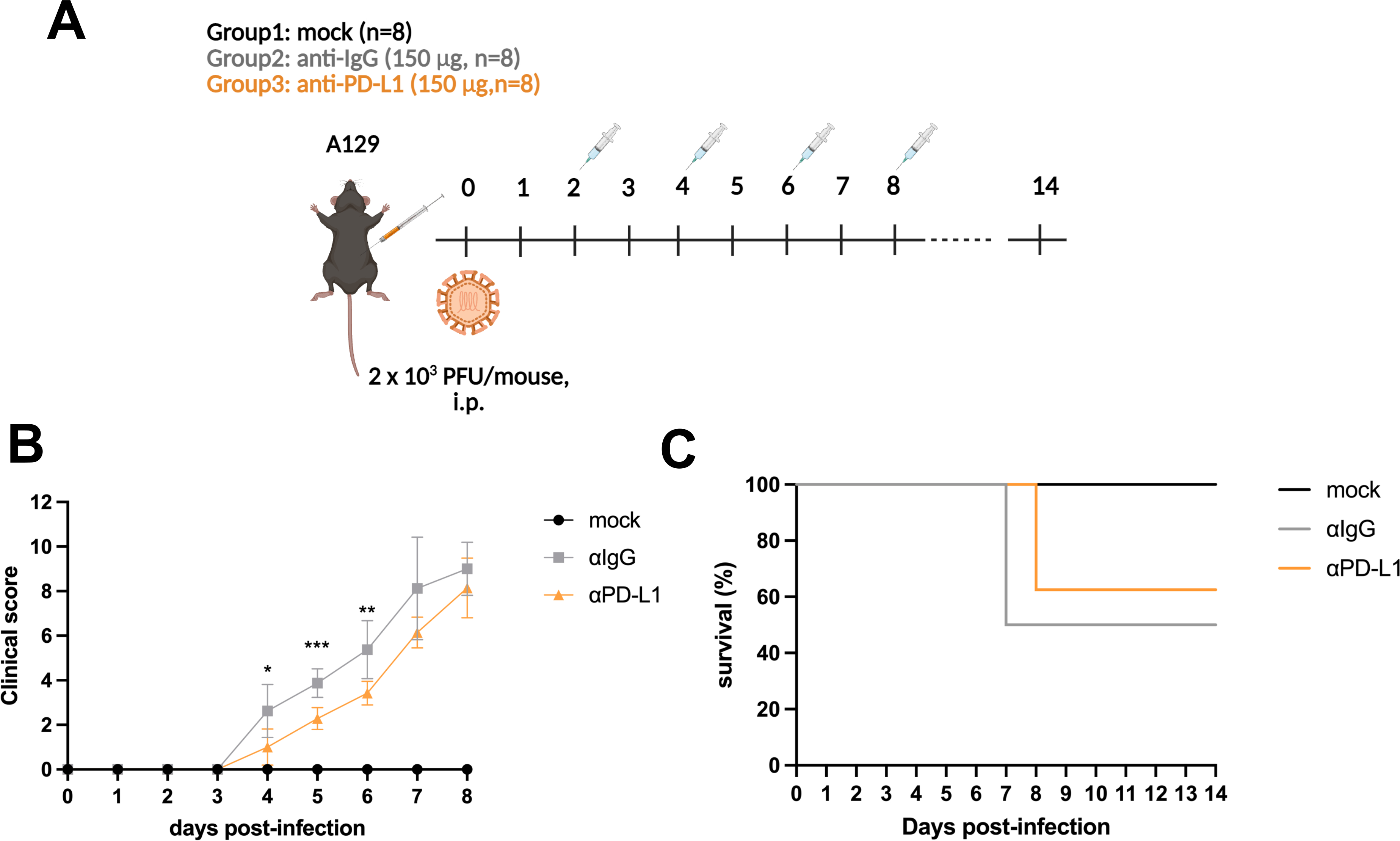

Supplement: S4 Fig — (A) Antibody treatment and viral challenge scheme for the A129 model for survival. Male A129 mice (n = 8/group) were i.p. inoculated with 2 × 10³ PFU of ZIKV and subsequently i.p. treated with 150 μg of either IgG control or anti-PD-L1 antibody on 2, 4, 6, and 8 dpi. Body weight changes in mice over a 14-day period were observed. The figure was created using BioRender (https://BioRender.com). (B) Clinical scores of A129 mice. The results are shown as the mean ± SD of three independent experiments. Statistical analyses were performed with two-way ANOVA (*P < 0.05; **P < 0.01; ***P < 0.001). (C) Survival rate of mice. Mice that lost more than 20% of their basal weight were euthanized. (TIF) [file ppat.1013457.s004.tif]

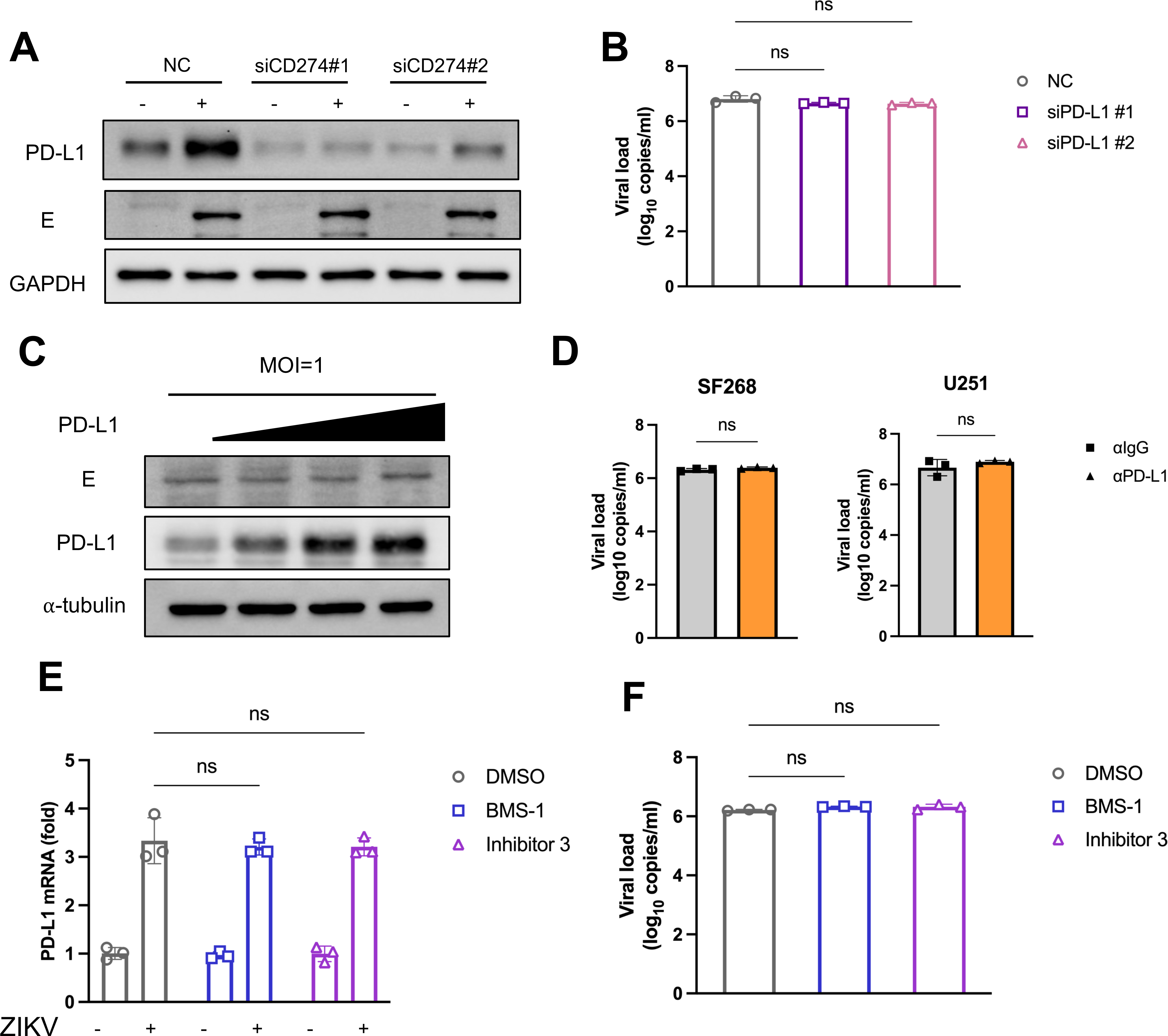

Supplement: S5 Fig — (A) U251 cells were transfected with control or PD-L1 siRNA for 24 hours, followed by ZIKV infection for 48 hours. Protein levels of PD-L1 and E protein were assessed by Western blotting with GAPDH as a loading control. (B) ZIKV RNA levels in the supernatant of infected cells were detected by one-step RT-qPCR. Statistical analyses were performed with one-way ANOVA. (C) SF268 cells were transfected with increasing dose of PD-L1. Protein levels of PD-L1 and E protein were assessed by Western blotting with ⍺-tubulin as a loading control. Results are representative of three independent experiments. (D) SF268 and U251 cells were infected with ZIKV at an MOI of 1. After 1 hour, the medium was replaced with 2% DMEM containing either IgG control (αIgG) or anti-PD-L1 (αPD-L1; Azeto) at 2 µg/ml. Viral RNA levels in the supernatant were quantified by one-step RT-qPCR at 24 hpi. Statistical significance was determined using Student’s t-test. (E) SF268 cells were infected with ZIKV at an MOI of 1. After a 1-hour incubation, the medium was replaced with 2% DMEM containing either PD-1/PD-L1 inhibitor 1 (BMS-1, 1 μM) or PD-1/PD-L1 inhibitor 3 (2 μM). Cell lysates were collected at 48 hpi. The mRNA levels of PD-L1 were assessed using RT-qPCR. GAPDH was used as a housekeeping control. Data are presented as the mean ± SD of three independent experiments. Statistical analyses were performed by two-way ANOVA. (F) ZIKV RNA levels in the supernatant of infected cells were detected by one-step RT-qPCR. Statistical analyses were performed with one-way ANOVA. (TIF) [file ppat.1013457.s005.tif]

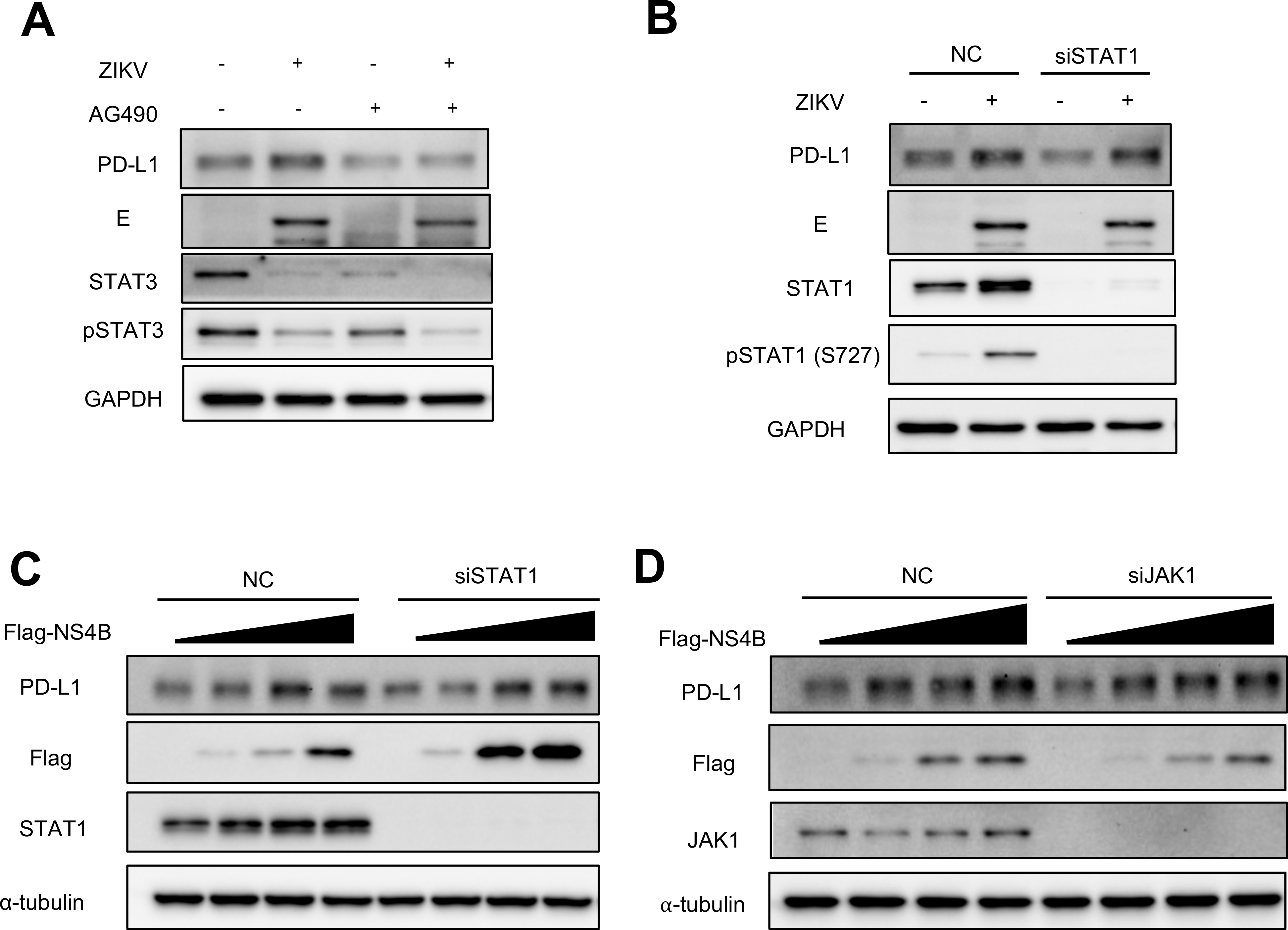

Supplement: S6 Fig — (A) Effect of AG490 on ZIKV-induced PD-L1 expression. SF268 cells were infected with ZIKV at an MOI of 1 for 24 hours and then treated with 50 μM AG490 for 12 hours. Western blot analysis of PD-L1, STAT3, pSTAT3 and ZIKV E protein in SF268 cells was performed with GAPDH as a loading control. Effect of STAT1 knockdown on PD-L1 expression induced by ZIKV (B) and NS4B (C). Western blot analysis of PD-L1, STAT1, ZIKV E/Flag-NS4B and GAPDH/α-tubulin expression levels in control or STAT1-silenced SF268 cells was performed. (D) Effect of JAK1 knockdown on NS4B-induced PD-L1 expression. Western blot analysis of PD-L1, JAK1, Flag-NS4B and α-tubulin expression levels in control or JAK1-silenced SF268 cells was performed. (TIF) [file ppat.1013457.s006.tif]

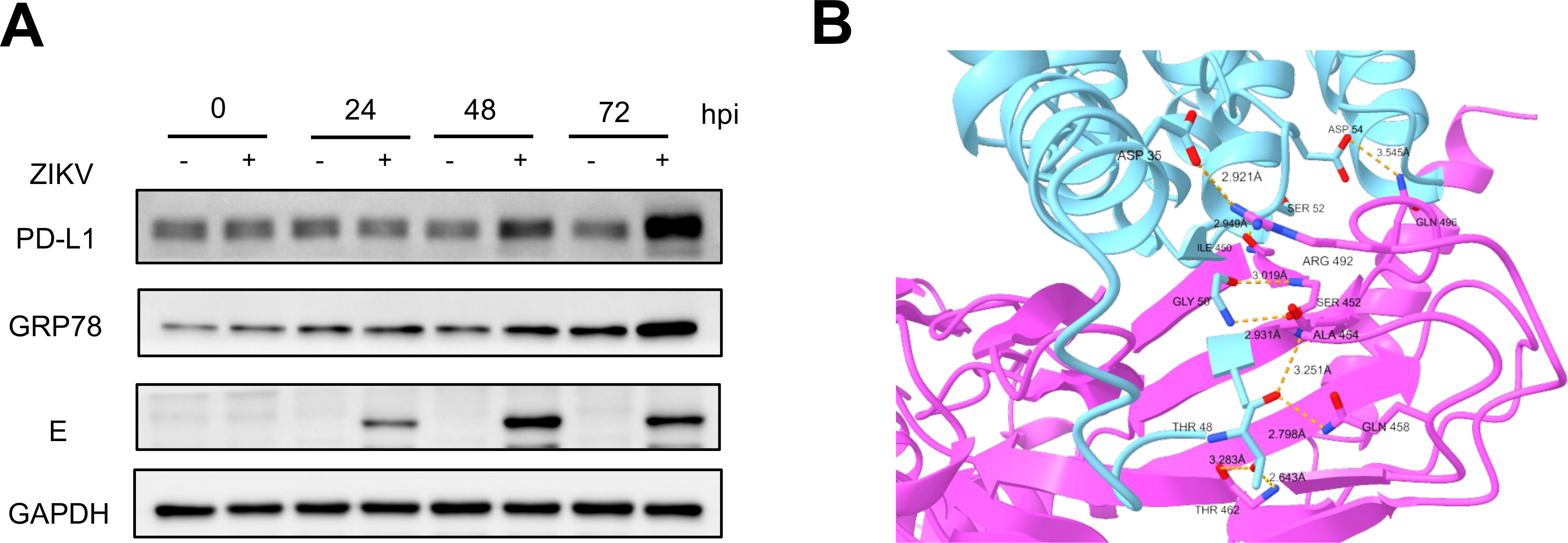

Supplement: S7 Fig — (A) Western blot analysis of PD-L1, GRP78 and ZIKV E protein in SF268 cells after ZIKV infection for 24, 48 and 72 hours. (B) AlphaFold analysis of the specific interaction domain and key amino acid site in both NS4B and GRP78. Blue: NS4B, pink: GRP78. (TIF) [file ppat.1013457.s007.tif]
